# Stem cell-based scaffolds using dental pulp and adipose-derived stem cells in periodontal regeneration: Evidence from preclinical or clinical studies

**DOI:** 10.6026/973206300222015

**Published:** 2026-04-30

**Authors:** Manimekalai Muthusamy, Beena George, Divya Alexander, Siladitya Sen, Suma Janya, Bindu Devanahalli Rajanna

**Affiliations:** 1 Department of Periodontics, Chettinad Institute of Higher Education and Research, Rajiv Gandhi Salai, Kelambakkam, Chennai, Tamil Nadu, India; 2 Department of Oral Pathology and Microbiology, Sree Mookambika Institute of Dental Sciences, Padanilam, Kulasekharam 629161, Tamil Nadu, India; 3 Department of Periodontics, Sri Rajiv Gandhi College of Dental Sciences and Hospital, Cholanagar, Bengaluru pin code 560032, Karnataka, India; 4 Department of Dentistry, JIMSH, K. P. Mondal Road, Buita, Budge Budge, Kolkata - 700137, West Bengal, India; 5 Department of Prosthodontics, RUAS (Ramaiah university of applied sciences), Msrit Edu campus New BEL Road, Bengaluru - 560064, Karnataka, India; 6 Department of Periodontology, Dayanada Sagar College of Dental Sciences, Kumaraswamy Layout, Bengaluru, Karnataka 560111, India

**Keywords:** Periodontal regeneration, stem cell-based scaffolds, dental pulp stem cells (DPSC), adipose-derived stem cells, tissue engineering, periodontal tissue engineering, alveolar bone regeneration, preclinical models, clinical applications

## Abstract

Current periodontal therapies fail to predictably restore the complete architecture of alveolar bone, periodontal ligament and cementum lost due to periodontal disease. Stem cell-based scaffolds have emerged as a biological strategy to enhance coordinated periodontal regeneration through combined cellular and biomaterial approaches. Therefore, it is of interest to evaluate preclinical and clinical evidence on dental pulp stem cell and adipose-derived stem cell-based scaffolds in periodontal regeneration. Animal studies consistently demonstrate enhanced new bone formation, cementum-like tissue deposition, organized periodontal ligament-like fibers and improved vascularization compared to scaffold-only controls. Thus, we report reductions in probing depth, gains in clinical attachment and favorable safety profiles, although standardized protocols and long-term data remain limited.

## Background:

Periodontal disease is a chronic inflammatory condition that progressively destroys alveolar bone, periodontal ligament and cementum, ultimately leading to tooth loss [1]. Conventional regenerative approaches, including guided tissue regeneration and bone grafting, improve defect fill but do not consistently restore the complete structural and functional architecture of the periodontium [2]. Tissue engineering strategies integrating stem cells, scaffolds and signaling molecules aim to biologically reconstruct lost periodontal tissues rather than merely repair defects [3]. Mesenchymal stem cells have gained attention due to their self-renewal capacity, multilineage differentiation and immunomodulatory properties that support tissue regeneration [4]. Dental pulp stem cells exhibit strong osteogenic and odontogenic potential and demonstrate affinity for craniofacial tissues due to their neural crest origin [5]. Adipose-derived stem cells provide an abundant and minimally invasive source of multipotent cells with pronounced paracrine and angiogenic effects that enhance tissue repair [6]. When combined with biocompatible scaffolds, these cells can create a supportive three-dimensional microenvironment that promotes cell adhesion, proliferation and spatial organization of regenerated tissues [7]. Preclinical studies report structured bone formation, cementum-like deposition and periodontal ligament-like fiber orientation following stem cell-scaffold implantation [8]. Early clinical investigations indicate improvements in probing depth reduction and clinical attachment gain, suggesting translational potential [9]. Despite promising outcomes, heterogeneity in scaffold materials, cell preparation methods and evaluation protocols limits direct comparison across studies and hinders standardization. Therefore, it is of interest to critically evaluate the role of dental pulp stem cell- and adipose-derived stem cell-based scaffolds in periodontal regeneration using evidence from preclinical and clinical studies.

## Materials and Methods:

This study was conducted to evaluate preclinical and clinical studies investigating dental pulp stem cell- and adipose-derived stem cell-based scaffolds in periodontal regeneration. Electronic searches were performed in PubMed, Scopus and Web of Science for studies published between January 2020 and March 2025 and written in English. Search terms combined controlled vocabulary and free-text keywords related to "periodontal regeneration," "dental pulp stem cells," "adipose-derived stem cells," "mesenchymal stem cells," "scaffolds" and "tissue engineering." Eligible studies included in vivo animal experiments evaluating periodontal defect regeneration using stem cell- scaffold constructs and clinical studies assessing regenerative outcomes following stem cell-based scaffold therapy. In vitro studies, case reports without regenerative outcome assessment, non-periodontal applications and studies published before 2020 were excluded. Data extracted from eligible studies included publication year, stem cell source, scaffold composition, defect model or clinical indication, duration of follow-up, assessment modality, regenerative outcomes and reported safety parameters. A qualitative synthesis approach was adopted due to heterogeneity in study design, defect models, scaffold materials and outcome measures. Emphasis was placed on biologically relevant endpoints including new bone formation, cementum-like tissue deposition, periodontal ligament organization, vascularization, clinical attachment gain, probing depth reduction and safety outcomes. Quantitative pooling was not performed because of variability in methodologies and reporting standards across studies.

## Results:

A total of eligible animal and clinical studies were included in the qualitative synthesis. The majority of investigations were preclinical animal studies evaluating intrabony or critical-size periodontal defects treated with stem cell-based scaffold constructs. Dental pulp stem cell-loaded scaffolds consistently demonstrated moderate to high levels of new bone formation compared to scaffold-only and untreated controls. Histological analyses frequently reported cementum-like tissue deposition and organized periodontal ligament-like fiber orientation in DPSC-treated groups. Adipose-derived stem cell-based scaffolds showed significant enhancement in vascularization and soft tissue integration, with measurable increases in mineralized tissue area in experimental defects. Micro-CT and histomorphometric assessments across animal models revealed greater bone volume fraction and improved structural organization in stem cell-treated defects. Composite scaffolds combining natural polymers and bioceramics demonstrated improved mechanical stability and regenerative performance. Safety evaluations in animal models reported transient local inflammatory responses without evidence of immune rejection, infection, or tumorigenicity. Clinical studies were limited in number and primarily consisted of pilot and early-phase prospective trials with follow-up periods ranging from 6 to 12 months. Patients treated with DPSC- or ADSC-based scaffold constructs exhibited significant reductions in probing pocket depth and measurable gains in clinical attachment level compared to baseline values. Radiographic assessments demonstrated increased defect fill and improved bone density in treated sites. No serious adverse events or systemic complications were reported in clinical applications. Despite consistent positive trends, heterogeneity in scaffold design, cell processing protocols and outcome measurement limited direct comparison across studies. Overall, evidence from both preclinical and early clinical investigations indicates biologically relevant periodontal regeneration with favorable safety profiles, although long-term standardized clinical data remain limited. Table 1 (see PDF) shows that dental pulp stem cells are derived from permanent or deciduous pulp through minimally invasive procedures and demonstrate osteogenic, odontogenic and fibroblastic differentiation potential relevant to cementum and periodontal ligament regeneration, whereas adipose-derived stem cells are obtained from subcutaneous adipose tissue with minimally invasive harvesting and exhibit osteogenic, angiogenic and immunomodulatory capacities that support bone regeneration and vascularization. Table 2 (see PDF) demonstrates that natural polymer scaffolds provide rapid biodegradation with low to moderate mechanical support suitable for intrabony defects, synthetic polymers offer controlled degradation with moderate structural stability, bioceramics provide slow resorption with high mechanical strength for alveolar bone support and composite scaffolds enable tunable degradation and high structural integrity for complex periodontal defects. Table 3 (see PDF) indicates that rat models are primarily used for early bone formation assessment within 4-8 weeks, rabbit models evaluate alveolar bone volume over 6-10 weeks, dog models assess functional periodontal ligament regeneration in critical- size defects over 8-12 weeks and mini-pig models provide extended evaluation up to 24 weeks with high translational relevance due to anatomical similarity to humans. Table 4 (see PDF) demonstrates that DPSC-loaded scaffolds consistently achieve moderate to high new bone formation with frequent cementum-like tissue deposition and organized periodontal ligament-like fiber orientation, whereas scaffold-only controls exhibit limited mineralized tissue formation and disorganized attachment structures. Table 5 (see PDF) shows that ADSC-based scaffolds produce moderate new bone formation with markedly enhanced vascularization and improved soft tissue integration compared to limited regenerative response in controls, with inflammatory reactions reported as mild and transient. Table 6 (see PDF) indicates that clinical studies using DPSC-based scaffolds report significant probing depth reduction, clinical attachment level gains ranging approximately from 1 to 3 mm and radiographic evidence of increased defect fill within follow-up periods of 6 to 12 months. Table 7 (see PDF) demonstrates that ADSC-based clinical investigations report significant probing depth reduction, moderate clinical attachment gains and favorable soft tissue healing responses across small sample prospective studies with follow-up durations up to 12 months. Table 8 (see PDF) shows that objective imaging modalities including micro-CT, histomorphometry, periapical radiographs and CBCT consistently demonstrate increased bone volume fraction, higher mineralized tissue area and improved defect density following stem cell-based scaffold therapy. Table 9 (see PDF) indicates that safety evaluations across animal and human studies report only mild or transient local inflammation with no documented immune rejection, infection, or tumorigenicity. Table 10 (see PDF) demonstrates that animal studies consistently report positive regenerative outcomes, clinical evidence remains early-phase with limited long-term follow-up, protocol standardization is lacking and the overall level of evidence is moderate and evolving.

## Discussion:

Stem cell-based scaffold strategies demonstrate consistent regenerative potential in preclinical periodontal models and emerging translational benefit in early clinical studies. Animal investigations show reproducible enhancement of new bone formation, cementum-like deposition and organized periodontal ligament-like fiber orientation when DPSCs are combined with bioactive scaffolds [10]. These findings suggest that dental pulp stem cells contribute to structural regeneration of the periodontal attachment apparatus through osteogenic differentiation and tissue-specific integration. In contrast, ADSC-based constructs exhibit strong angiogenic and immunomodulatory effects, which appear to enhance vascularization and soft tissue healing within periodontal defects [11]. The synergistic interaction between stem cells and scaffold architecture is critical for spatial organization and functional tissue formation [12]. Natural polymer scaffolds support cellular attachment and biodegradation, whereas composite scaffolds provide enhanced mechanical stability for defect maintenance [13]. The presence of organized ligament-like fibers in DPSC-treated models indicates partial restoration of functional attachment rather than mere bones fill [14]. Enhanced vascular density observed in ADSC groups supports the concept that paracrine signaling contributes significantly to regenerative outcomes [15]. Clinical investigations, although limited in number and duration, report measurable reductions in probing depth and gains in clinical attachment within 6 to 12 months [16]. Radiographic and CBCT assessments demonstrate increased defect fill and improved mineral density at treated sites [17]. Importantly, no serious adverse events, immune reactions, or tumorigenic complications have been reported in available studies [18]. However, variability in scaffold composition, cell isolation protocols, defect morphology and outcome measurement limits comparability across trials [19]. The absence of standardized processing techniques and long-term follow-up restricts definitive conclusions regarding durability and predictability of regeneration [20].

## Conclusion:

Stem cell-based scaffolds utilizing dental pulp stem cells and adipose-derived stem cells demonstrate biologically relevant periodontal regeneration in preclinical models and promising clinical improvements in early human studies. Evidence supports enhanced new bone formation, organized periodontal ligament-like attachment, improved vascularization and favorable safety profiles compared to conventional regenerative approaches. However, standardized cell processing protocols, optimized scaffold design and long-term controlled clinical trials are required before routine clinical integration can be recommended.

## Advancement to knowledge:

Advancement to knowledge in this review lies in the integrated evaluation of biological performance, scaffold interaction, imaging-based outcomes and safety parameters specific to DPSCs and ADSCs in periodontal regeneration. By distinguishing structural regenerative effects from angiogenic and immunomodulatory contributions, this synthesis clarifies the differential biological roles of these two stem cell populations. The evidence supports biologically relevant periodontal regeneration, yet highlights the need for standardized, controlled and long-term clinical investigations to establish reproducible therapeutic protocols.
